# Supplementary material for: Approaches to describing inter-rater reliability of the overall clinical appearance of febrile infants and toddlers in the emergency department
Source: PeerJ. 2014 Nov 11;2:e651. doi: 10.7717/peerj.651 (PMC4230550; doi:10.7717/peerj.651)
Supplement: Appendix S1 [file peerj-02-651-s001.docx]

**Appendix 1. Selected R code to calculate AC1 (Not peer reviewed)**

# Input data

data_forR <- read.csv("~/PIRS/data_forR.csv")

#The original author was Dr. Pedro Emmanuel A. A. do Brasilemail: pedro.brasil@ipec.fiocruz.bremail: emmanuel.brasil@gmail.com

# The credit is all his, the errors all mine

# originally posted at https://stat.ethz.ch/pipermail/r-sig-epi/attachments/20120503/7c03297b/attachment.pl

AC1 <- function(table,conflev=0.95,N=Inf,print=TRUE){

if(dim(table)[1] != dim(table)[2]){

stop('The table should have the same number of rows and columns!')

}

n <- sum(table)

f <- n/N

pa <- sum(diag(table))/n # formula 18

q <- ncol(table) # number of categories

pkk <- diag(table)/n

pak <- sapply(1:q,function(i)sum(table[i,]))/n

pbk <- sapply(1:q,function(i)sum(table[,i]))/n

pik <- (pak + pbk)/2

pegama <- (sum(pik*(1-pik)))/(q-1)

gama <- (pa - pegama)/(1 - pegama) # AC1 statistics

# 2 raters special case variance

pkl <- table/n

soma <- 0;

for(k in 1:q){

for(l in 1:q){

soma <- soma + (pkl[k,l]*((1-(pik[k]+pik[l])/2)^2))

}

}

vgama <- ((1-f)/(n*(1-pegama)^2)) * (pa*(1-pa) - 4*(1-gama)*((1/(q-1))*sum(pkk*(1-pik)) - pa*pegama) + 4*((1-gama)^2) *((1/((q-1)^2))*soma - pegama^2))

epgama <- sqrt(vgama)# AC1 standard error

lcb <- max(0,gama - epgama*qnorm(1-(1-conflev)/2,0,1)) # lower confidencebound

ucb <- min(1,gama + epgama*qnorm(1-(1-conflev)/2,0,1)) # upper confidence bound

if(print==TRUE){

cat('Raw agreement:',pa,'Chance-independent agreement:',pegama,'\n')

cat('Agreement coeficient (AC1):',gama,'AC1 standard error:',epgama,'\n')

cat(conflev*100,'% Confidence Interval (AC1): (',lcb,',',ucb,')\n')

}

invisible(c(pa,pegama,gama,epgama,lcb,ucb))

}

inter1 <-table(impression1, impression3)

AC1(inter1)

x <- AC1(inter1,print=F)

print(x)

inter2 <-table(impression2 ,impression4)

x1 <- AC1(inter2,print=F)

print(x1)

intra1 <-table(impression1 ,impression2)

x2<- AC1(intra1, print=F)

print(x2)

intra2 <-table(impression3, impression4)

x3<-AC1(intra2 ,print=F)

print(x3)
