# Supplementary material for: Approaches to describing inter-rater reliability of the overall clinical appearance of febrile infants and toddlers in the emergency department
Source: PeerJ. 2014 Nov 11;2:e651. doi: 10.7717/peerj.651 (PMC4230550; doi:10.7717/peerj.651)
Supplement: Appendix S2 [file peerj-02-651-s002.docx]

**Appendix 2. Selected Stata code for data management and generating graphs (Not peer reviewed)**

//

//

//Sacramento

//

//PIRS

//

//clear all

import delimited C:\Users\8core\Downloads\pirs.mer, varnames(1) asdouble clear

format %12.0f accountnumber

sort accountnumber

gen id2 =_n

isid id2

rename stexamtime firststexamtime

rename ndexamtime secondndexamtime

// code to correct data entry errors ommitted

gen time1 =date1 + firststexamtime

gen time2 =date2 + secondndexamtime

gen double t1 = clock(time1 , "MDYhm")

gen double t2 = clock(time2 , "MDYhm")

gen interval = (t2-t1) /60000

label var t1 "time/date first exam"

label var t2 "time/date second exam"

label var interval " Minutes between two exams"

gen dob = date(dateofbirth , "MDY" )

format %td dob

list dateofbirth dob

label var dob "DoB"

label var initialimpression4 "Second MD Impression after exam"

label var initialimpression3 "Second MD Gestalt Impression"

label var initialimpression1 "First MD Gestalt Impression"

label var initialimpression2 "First MD Impression after exam"

label var tylenolbeforeexam "Any antipyretic before first exam"

label var tylenolintermediate "Any antipyretic after first and before second exam"

//Check for discrepancies

list acc if interval >240

list acc if interval <0

list acc if disposition ==""

duplicates tag studyid , gen(tag)

list accountnumber studyid if(tag) ,sepby(studyid )

duplicates tag mrec ,gen(tag2)

sort mrec

list accountnumber mrec studyid if (tag2) ,sepby (mrec)

list accountnumber if tylenolbeforeexam ==""

list accountnumber date1 firststexamtime date2 secondndexamtime if tylenolintermediate ==""

// Turning string to labelled coded variables as needed

gen admit =.

replace admit =1 if disposition =="Admit"

replace admit =0 if disposition =="Discharge"

label var admit "Admitted =1"

label def dispo 0 "Discharged" 1 "Admitted"

//

gen dos =date(date,"MDY")

format %td dos

label var dos "Date of Service"

//

///

// CREATING THE DATASET FOR FIGURE 1, PATIENT FLOW

//

//

cap drop agedays

cap drop age

gen agedays =(dos-dob)

label var agedays "Age in days"

gen age =(dos-dob)/30.25

label var age "Age in months"

cap drop tag1

drop if agedays >730

duplicates tag mrec , gen(tag1)

tab tag1

duplicates drop mrec ,force

isid mrec

//

// Para1 1 infant characteristics /age/gender/dispostion

//Table 1 Provider Range in years, range in time

//

//para1

tabstat age ,stat(med iqr p25 p75)

tab sex

tab admit

// Table 1 list of diagnoses

// Provider pairs

cap drop pair_type1

gen pair_type1 =0

replace pair_type1 =1 if regexm(providertype, "Attending" )==1 & regexm(providertype2, "Attending" )==1

replace pair_type1 =1 if regexm(providertype, "Attending" )==1 & regexm(providertype2, "R4" )==1

replace pair_type1 =1 if regexm(providertype, "Attending" )==1 & regexm(providertype2, "PA-C" )==1

replace pair_type1 =1 if regexm(providertype2, "Attending" )==1 & regexm(providertype, "PA-C" )==1

replace pair_type1 =1 if regexm(providertype2, "Attending" )==1 & regexm(providertype, "R4" )==1

replace pair_type1 =1 if regexm(providertype2, "PA-C" )==1 & regexm(providertype, "R4" )==1

replace pair_type1 =1 if regexm(providertype, "PA-C" )==1 & regexm(providertype2, "R4" )==1

label var pair_type1 "Both observers were Attending R4 or PA-C"

cap drop pair_type2

gen pair_type2 =0

replace pair_type2 =1 if regexm(providertype,"R1") ==1 & regexm(providertype2,"R4")==1

replace pair_type2 =1 if regexm(providertype,"R2") ==1 & regexm(providertype2,"Attending")==1

replace pair_type2 =1 if regexm(providertype,"R1") ==1 & regexm(providertype2 ,"PA-C")==1

replace pair_type2 =1 if regexm(providertype,"R3") ==1 & regexm(providertype2,"Attending")==1

replace pair_type2 =1 if regexm(providertype2,"R1") ==1 & regexm(providertype ,"R4")==1

replace pair_type2 =1 if regexm(providertype2,"R2") ==1 & regexm(providertype ,"Attending")==1

replace pair_type2 =1 if regexm(providertype2,"R1") ==1 & regexm(providertype ,"PA-C")==1

replace pair_type2 =1 if regexm(providertype2,"R3") ==1 & regexm(providertype,"Attending")==1

label var pair_type2 "Difference >2 yrs in experience between raters"

//

gen antipyretic_home = .

replace antipyretic_home =1 if tylenolbeforeexam =="Yes"

replace antipyretic_home =0 if tylenolbeforeexam =="No"

gen antipyretic_between = .

replace antipyretic_between =1 if tylenolintermediate =="Yes"

replace antipyretic_between =0 if tylenolintermediate =="No"

label var antipyretic_home "Antipyretic before presentation"

label var antipyretic_between "Antipyretic between exams"

cap label drop yesno

label def yesno 0 "No" 1 "Yes"

label val antipyretic_home yesno

label val antipyretic_between yesno

cap label drop appear

label def appear 0 "Well Appearing" 1 "Not Sure" 2 "Ill Appearing"

forval a=1(1)4 {

cap drop impression`a'

gen impression`a' =.

replace impression`a' = 0 if initialimpression`a' =="Well Appearing"

replace impression`a' = 1 if initialimpression`a' =="Not Sure or Equivocal"

replace impression`a' = 2 if initialimpression`a' =="Ill Appearing"

label val impression`a' appear

}

label var impression1 "First MD Gestalt Impression"

label var impression2 "First MD Impression after exam"

label var impression3 "Second MD Gestalt Impression"

label var impression4 "Second MD Impression after exam"

//

// Differences

//

cap drop dif_inter_gestalt

cap drop dif_inter_after

cap drop dif intra*

cap label drop agree

label def agree 0 "Agree" 1 "Rater 1 one categories more ill" 2" Rater 1 two categories more ill" -1 "Rater1 one categories less ill" -2" Rater1 two categories less ill"

gen dif_inter_gestalt = impression1 - impression3

label var dif_inter_gestalt "Difference in Gestalt assessment"

gen dif_inter_after = impression2- impression4

label var dif_inter_after "Difference in assessment after full exam"

gen dif_intra1 = impression1-impression2

gen dif_intra2 = impression3-impression4

label val dif_inter_after agree

label val dif_inter_gestalt agree

//

//

// Variables for looking at things that affect agreement

//

cap drop s_gestalt

cap drop s_after

gen s_gestalt = 0

replace s_gestalt =1 if dif_inter_gestalt==0

gen s_after =0

replace s_after =1 if dif_inter_after==0

label var s_after "1 if agreed after examining otherwise 0 "

label var s_gestalt "1 if agreed Gestalt otherwise 0"

cap drop agelt2

gen agelt2 =0

replace agelt2 =1 if age <=2

//

//

cap drop dx

cap drop dx2

cap drop dx_simple

cap label drop dx

cap label drop dxs

gen dx =.

label def dx 1 "Pneumonia" 2" UTI/Pyelonephritis" 3 "Bronchiolitis" 4 "Otitis media" 5 " Croup" 6 "Gastroenteritis" 7 "Cellulitis" 8 "Sepsis No focus" 9 "URI" 10 "Herpangina" 12 "Febrile illness NOS" 13 "Bacteremia" 14 "Varicella" 15 "Febrile Seizure" 20 "Non infective" 22 "Other Febrile illnesses" 11 "Pharyngitis"

replace diagnosis=lower(diagnosis)

replace dx = 1 if regexm(diagnosis , "pneum")

replace dx = 2 if regexm(diagnosis , "UTI") | regexm(diagnosis,"uti") | regexm(diagnosis,"pyel")

replace dx = 3 if regexm(diagnosis , "bronchiolitis")

replace dx = 4 if regexm(diagnosis , "media")

replace dx = 5 if regexm(diagnosis , "croup")

replace dx = 6 if regexm(diagnosis , "gastroenteritis") |regexm(diagnosis, "enteritis" )| regexm(diagnosis, "age")

replace dx = 7 if regexm(diagnosis , "cellulitis") |regexm(diagnosis ,"sepsis")

replace dx= 7 if regexm(diagnosis,"lower lip infection") |regexm(diagnosis, "skin infection")

replace dx = 8 if regexm(diagnosis , "croup")

replace dx =3 if regexm(diagnosis, "upper respiratory infection, with bronchospasm")

replace dx =9 if regexm(diagnosis,"uri")

replace dx=9 if regexm(diagnosis, "upper respiratory inpression")

replace dx =10 if regexm(diagnosis, "herpang" )

replace dx = 12 if regexm(diagnosis , "febrile illness") |regexm(diagnosis , "viral illness") |regexm(diagnosis ,"viral syndrome") & dx ==.

replace dx =12 if diagnosis=="fever." |diagnosis =="fever"

replace dx = 9 if regexm(diagnosis,"upper respiratory infection")

replace dx =11 if regexm(diagnosis, "pharyngitis")

replace dx =13 if regexm(diagnosis,"bacteremia")

replace dx=14 if regexm(diagnosis, "varicella")

replace dx=15 if regexm(diagnosis, "seizure")

replace dx =3 if diagnosis=="reactive airway disease." |diagnosis =="rsv."

replace dx=20 if diagnosis=="diaper rash."|regexm(diagnosis, "well child visit")|regexm(diagnosis, "factitious")

replace dx=6 if regexm(diagnosis, "gi infection")

replace dx =22 if dx==.

label val dx dx

gen dx_simple = dx

replace dx_simple = 12 if dx_simple ==22

replace dx_simple = 8 if dx_simple ==13

label def dxs 1 "Pneumonia" 2" UTI/Pyelonephritis" 3 "Bronchiolitis" 4 "Otitis media" 5 " Croup" 6 "Gastroenteritis" 7 "Cellulitis" 8 "Sepsis/Bacteremia" 9 "URI" 10 "Herpangina" 12 "Febrile illness NOS" 13 "Bacteremia" 14 "Varicella" 15 "Febrile Seizure" 20 "Non infective" 22 "Other Febrile illnesses" 11 "Pharyngitis"

label val dx_simple dxs

//

//

cap drop prvd1

cap drop prvd2

cap label drop prvd

gen prvd1 =.

replace prvd1 =1 if providertype =="R1" |providertype=="Rotating Resident"

replace prvd1 = 2 if providertype=="R2"

replace prvd1 = 3 if providertype=="R3"

replace prvd1 = 4 if providertype=="R4"

replace prvd1 = 5 if providertype=="PA-C"

replace prvd1 = 5 if providertype=="PA-C/NP"

replace prvd1 = 6 if providertype=="Attending Phys."

gen prvd2 =.

replace prvd2 =1 if providertype2 =="R1" |providertype=="Rotating Resident"

replace prvd2 = 2 if providertype2=="R2"

replace prvd2 = 3 if providertype2=="R3"

replace prvd2 = 4 if providertype2=="R4"

replace prvd2 = 5 if providertype2=="PA-C"

replace prvd2 = 5 if providertype2=="PA-C/NP"

replace prvd2 = 6 if providertype2=="Attending Phys."

label def prvd 1 "Intern/rotator" 2 " EM-R2" 3 "EM-R3" 4 "EM-R4" 5 "PA-C" 6 "Attending"

label val prvd1 prvd

label val prvd2 prvd

//

// Figure 2

sunflower prvd1 prvd2 , binar(2.25) binwidth(0.25) scheme(s2mono) // in editor us grey16 for background and manully add labels

//Figure 3 after executing command try runnnng macro to automate graph cleanup

catplot dif_inter_gestalt ,percent scheme(s1mono)

catplot dif_inter_after ,percent scheme(s1mono)

gen interval10 = interval /10

label var interval10 "Number of 10 minute intervals between evaluations"

// create data for intrarater graph

//Sacramento 7/10/13

import delimited C:\Users\8core\Documents\PIRS\data_for_single_intra_graph.csv ,clear

save i_graph1.dta, replace

append using i_graph1.dta

drop dif_intra2

rename dif_intra1 intra_dif

cap label drop agree

label def agree 0 "Agree" 1 "Rater 1 one categories more ill" 2" Rater 1 two categories more ill" -1 "Rater1 one categories less ill" -2" Rater1 two categories less ill"

label val intra_dif agree

catplot intra_dif ,percent scheme(s1mono)

//

// import main file, export rating and provider

use C:\Users\8core\Documents\PIRS\pirs.dta

export delimited mrec age interval impression1 prvd1 using "C:\Users\8core\Documents\PIRS\data_seniority_gestalt_A.csv",nolabel replace

export delimited mrec age interval impression3 prvd2 using "C:\Users\8core\Documents\PIRS\data_seniority_gestalt_B.csv",nolabel replace

clear

import delimited C:\Users\8core\Documents\PIRS\data_seniority_gestalt_A.csv, clear

rename impression1 imp_G

save gestalt_A.dta, replace

clear

import delimited C:\Users\8core\Documents\PIRS\data_seniority_gestalt_B.csv, clear

rename impression3 imp_G

save gestalt_B.dta, replace

use gestalt_A.dta

append using gestalt_B.dta

cap label drop appear

label def appear 0 "Well Appearing" 1 "Not Sure" 2 "Ill Appearing"

label val imp_G appear

cap drop pv

cap label drop prvd

gen pv =prvd1

replace pv = prvd2 if pv==.

label def prvd 1 "Intern/rotator" 2 " EM-R2" 3 "EM-R3" 4 "EM-R4" 5 "PA-C" 6 "Attending"

label val pv prvd

drop prvd1 prvd2

save data_for_seniority_effect_graph.dta, replace

gr bar cat0 cat1 cat2 ,stack over(pv) legend(row(1)) scheme(s1mono) intensity(80)
