# Supplementary material for: Approaches to describing inter-rater reliability of the overall clinical appearance of febrile infants and toddlers in the emergency department
Source: PeerJ. 2014 Nov 11;2:e651. doi: 10.7717/peerj.651 (PMC4230550; doi:10.7717/peerj.651)
Supplement: Appendix S3 [file peerj-02-651-s003.docx]

**Appendix 3.**

This simulates what would have happened if the first rater had been the second rater and vice versa.

//Sacramento

//Permutation testing

//

//

cap drop random_*

cap drop R_imp*

cap drop minK

cap drop maxK

cap drop kappa*

forval i =1(1)500{

set seed `i'`i'

gen random_`i' =2*(runiform())

list random_`i' in 1

gen R_imp_first_`i' = impression1 if random_`i' <=1

gen R_imp_second_`i' = impression1 if R_imp_first_`i'==.

replace R_imp_first_`i' = impression3 if R_imp_first_`i' ==.

replace R_imp_second_`i' =impression3 if R_imp_second_`i'==.

kap R_imp_first_`i' R_imp_second_`i' , w(w2)

gen kappa_`i' =r(kappa)

}

egen minK= rowmin(kappa_*)

egen maxK= rowmax(kappa_*)
