# Supplementary material for: Approaches to describing inter-rater reliability of the overall clinical appearance of febrile infants and toddlers in the emergency department
Source: PeerJ. 2014 Nov 11;2:e651. doi: 10.7717/peerj.651 (PMC4230550; doi:10.7717/peerj.651)
Supplement: Appendix S4 [file peerj-02-651-s004.docx]

**Appendix 4.**  Additional measures of agreement demonstrating the number of results that could be obtained in an inter rater reliability study. (Expanded Table 5) Output from Agreestat.

| **Inter rater Gestalt** |  |  |
| --- | --- | --- |
| Unweighted | Linear weighting | Quadratic weighting |
| Cohen’s κ 0.118  AC1 0.550  Scott’s π 0.118  Krippendorf’s α 0.120  Brennan-Prediger 0.462  Percent 64.1% | Cohen’s κ s 0.177  AC2 0.601  Scott’s π 0.177  Krippendorf’s α f 0.179  Brennan-Prediger 0.406  Percent 73.6 | Cohen’s κ s 0.220  AC2 0.6345  Scott’s π 0.2197  Krippendorf’s α 0.2221  Brennan-Prediger 0.3491  Percent 78.3 |
| **Inter rater after exam** |  |  |
| Unweighted | Linear weighting | Quadratic weighting |
| Cohen’s κ 0.235  AC1 0.6546  Scott’s π 0.2163  Krippendorf’s α 0.2188  Brennan-Prediger 0.5754  Percent 71.7% | Cohen’s κ 0.2609  AC2 0.6716  Scott’s π 0.2608  Krippendorf’s α 0.263  Brennan-Prediger 0.4834  Percent 77.0 | Cohen’s κ 0.2891  AC2 0.6825  Scott’s π 0.28896  Krippendorf’s α 0.2912  Brennan-Prediger 0.33915  Percent 79.7 |
| **First rater with self** |  |  |
| Unweighted | Linear weighting | Quadratic weighting |
| Cohen’s κ 0.695  AC1 0.851  Scott’s π 0.6948  Krippendorf’s α 0.6958  Brennan-Prediger 0.8208  Percent 88.1 | Cohen’s κ 0.7773  AC-2 0.8926  Scott’s π 0.7771  Krippendorf’s α 0.7778  Brennan-Prediger0.8373  Percent 92.8 | Cohen’s κ 0.8325  AC-2 0.91988  Scott’s π 0.8325  Krippendorf’s α 0.8329  Brennan-Prediger 0.85377  Percent 95.1 |
| **Second rater with self** |  |  |
| Unweighted | Linear weighting | Quadratic weighting |
| Cohen’s κ 0.6717  AC1 0.8386  Scott’s π 0.6709  Krippendorf’s α 0.67193  Brennan-Prediger 0.80555  Percent 87.0 | Cohen’s κ 0.7346  AC-2 0.8713  Scott’s π 0.7343  Krippendorf’s α 0.7351  Brennan-Prediger 0.8055  Percent 91.3 | Cohen’s κ 0.7773  AC-2 0.8931  Scott’s π 0.7773  Krippendorf’s α 0.77797  Brennan-Prediger 0.8056  Percent 93.5 |
