# Supplementary material for: Approaches to describing inter-rater reliability of the overall clinical appearance of febrile infants and toddlers in the emergency department
Source: PeerJ. 2014 Nov 11;2:e651. doi: 10.7717/peerj.651 (PMC4230550; doi:10.7717/peerj.651)
Supplement: Appendix S5 [file peerj-02-651-s005.docx]

**Appendix 5.** Logistic regression models: fit calibration and classification characteristics

//stata version 12/13

logit s_gestalt interval10 age , or

estat gof

linktest

estat ic

estat class

logit s_gestalt interval10 age , or

fitstat

collin interval10 age

predict p

gen index =_n

predict resid_std , rstand

scatter resid p

scatter p index

scatter resid_std index ,mlabel(interval)

predict dev, dev

scatter dev index

scatter dev index ,mlabel (interval) caption(Deviance residual: Index plot labelled by interval betyween evaluations in minutes)

predict hat , hat

scatter hat index , mlabel(interval) caption(Leverage: Index plot labelled with interval between raters)

Predicted probability versus standardized residuals Index plot or residuals labeled with time

Similar information in deviance index plot Leverage Index shows effect of time outliers on agreement

Predicted probability versus standardized residuals Index plot or residuals labeled with time

Similar information in deviance index plot Leverage Index shows effect of time outliers on agreement
