# Supplementary material for: Approaches to describing inter-rater reliability of the overall clinical appearance of febrile infants and toddlers in the emergency department
Source: PeerJ. 2014 Nov 11;2:e651. doi: 10.7717/peerj.651 (PMC4230550; doi:10.7717/peerj.651)
Supplement: Supplemental Information 6 — Description of variables and labels [file peerj-02-651-s006.pdf]

obs: 159
vars: 59 18 Jul 2014 00:17
size: 88,563 (_dta has notes)

variable name	storage type	display format	value label	variable label
firststexamtime	str5	%9s		1st Exam Time
secondndexamt~e	str5	%9s		2nd Exam Time
audit	str3	%9s		Audit
auditorname	str16	%16s		Auditor Name
date	str10	%10s		Date
date1	str10	%10s		Date 1
date2	str10	%10s		Date 2
dateofbirth	str10	%10s		Date of Birth
diagnosis	str106	%106s		Raw diagnosis
disposition	str9	%9s		Disposition
dispotime	str10	%10s		
examiner1	str14	%14s		Examiner 1
examiner2	str15	%15s		Examiner 2
initialimpres~1	str21	%21s		First MD Gestalt Impression
initialimpres~2	str21	%21s		First MD Impression after exam
initialimpres~3	str21	%21s		Second MD Gestalt Impression
initialimpres~4	str21	%21s		Second MD Impression after exam
providertype	str17	%17s		Provider Type
providertype2	str17	%17s		Provider Type 2
raname	str19	%19s		RA Name
sex	str6	%9s		Sex
studyid	str5	%9s		Study ID
tylenolbefore~m	str3	%9s		Any antipyretic before first exam
tylenolinterm~e	str3	%9s		Any antipyretic after first and before second exam
id2	float	%9.0g		
time1	str20	%20s		Date/Time first exam -MASKED
time2	str20	%20s		Date/Time first exam -MASKED
t1	double	%10.0g		Masked for privacy time/date first exam
t2	double	%10.0g		Masked for privacy time/date second exam
interval	float	%9.0g		Minutes between two exams
dob	float	%td		Masked =99 for privacy
admit	float	%9.0g		Admitted =1
dos	float	%td		Date of Service
agedays	float	%9.0g		Age in days
age	float	%9.0g		Age in months
pair_type1	float	%9.0g		Both observers were Attending R4 or PA-C
pair_type2	float	%9.0g		Difference >2 yrs in experience between raters
antipyretic_h~e	float	%9.0g	yesno	Antipyretic before presentation
antipyretic_b~n	float	%9.0g	yesno	Antipyretic between exams
impression1	float	%14.0g	appear	First MD Gestalt Impression
impression2	float	%14.0g	appear	First MD Impression after exam
impression3	float	%14.0g	appear	Second MD Gestalt Impression
impression4	float	%14.0g	appear	Second MD Impression after exam
dif_inter_ges~t	float	%32.0g	agree	Difference in Gestalt assessment
dif_inter_after	float	%32.0g	agree	Difference in assessment after full exam
dif_intra1	float	%9.0g		Difference in first Intra raters' evaluations
dif_intra2	float	%9.0g		Difference in first Intra raters' evaluations
s_gestalt	float	%9.0g		1 if agreed Gestalt otherwise 0
s_after	float	%9.0g		1 if agreed after examining otherwise 0
dx	float	%23.0g	dx	Grouped clinical diagnosis at disposition
dx_simple	float	%23.0g	dxs	Simplified grouped clinical diagnosis at disposition
prvd1	float	%14.0g	prvd	Coded provider level first examiner
prvd2	float	%14.0g	prvd	Coded provider level second examiner
interval10	float	%9.0g		Number of 10 minute intervals between evaluations
pr1	float	%9.0g	pr_summary	Provider 1 , 1 =pgy1/2 2=pgy3/4 3 =MLP 4+attending
pr2	float	%9.0g	pr_summary	Provider 2 , 1 =pgy1/2 2=pgy3/4 3 =MLP 4+attending
dif_pr	float	%9.0g		Diference in providers -pr coding scheme
dif_prvd	float	%9.0g		Diference in providers -prvd coding scheme
id_masked	float	%9.0g		
